# Phase separation of Dri1 contributes to heterochromatin formation in *Schizosaccharomyces pombe*

**DOI:** 10.17912/micropub.biology.000559

**Published:** 2022-04-19

**Authors:** Hyoju Ban, Wenqi Sun, Yong Chen, Fei Li

**Affiliations:** 1 Department of Biology, New York University, New York, NY 10003, USA.; 2 Shanghai Institute of Biochemistry and Cell Biology, Center for Excellence in Molecular Cell Science, Chinese Academy of Sciences, Shanghai, 200031, China.; 3 University of Chinese Academy of Sciences, Beijing 100049, China.

## Abstract

The RNA binding protein Dri1 facilitates heterochromatin assembly via the RNAi pathway and histone deacetylases (HDAC). Dri1 contains an intrinsically disordered region (IDR) and three zinc fingers at its C-terminus, which are important for its role in heterochromatin silencing. Both IDR and zinc fingers have been implicated in mediating liquid-liquid phase separation (LLPS). In this study, we investigated the phase separation properties of Dri1. We observed that Dri1 undergoes phase separation *in vitro* . Dri1 also exhibits liquid-like behavior *in vivo* . Combined with our previous findings, our data support a model in which the phase-separated condensates formed by Dri1 may help recruit RNAi components and HDAC to mediate heterochromatin assembly.

**Figure 1. Dri1 exhibits liquid-like phase-separated behavior in vitro and in vivo.**
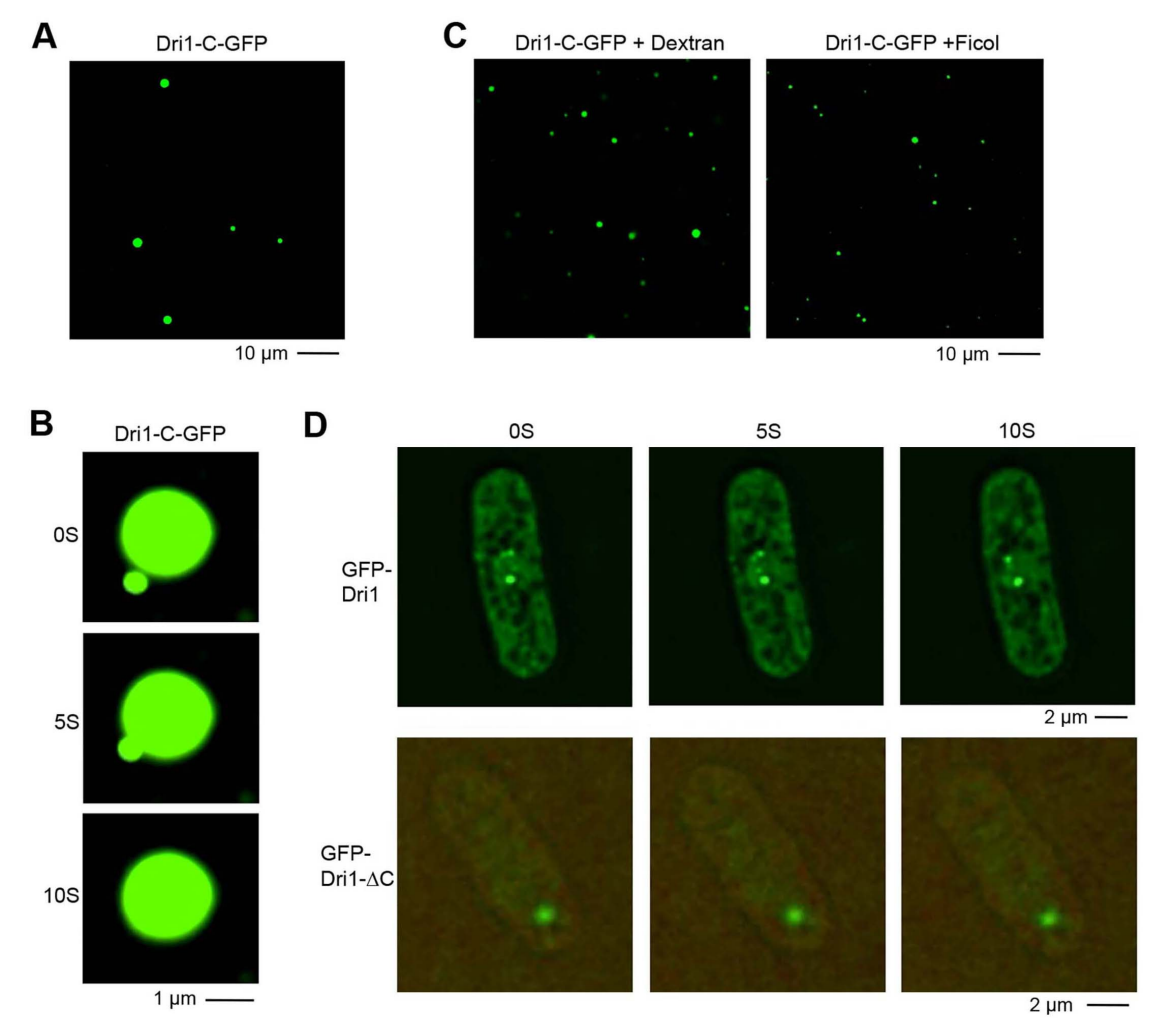
A. Fluorescence microscopy images of liquid-like droplets formed by Dri1-C-GFP at 150 mM NaCl. B. Time-lapse fluorescence microscopy of droplet-like structures formed by Dri1-C-GFP at 150 mM NaCl. Note: the spherical droplets in the solution can flow and fuse. C *.* Fluorescence microscopy images of liquid-like droplets form by Dri1-C-GFP in the presence of 10% Dextran or Ficoll at 150 mM NaCl. D. Top: Lowly overexpressed GFP-Dri1 induces liquid-like condensates *in vivo* . Time-lapse fluorescence microscopy analysis of a typical live cell expressing GFP-Dri1. Lower: Time-lapse fluorescence microscopy of a typical live cell expressing GFP-Dri1-∆C. Cells with lowly overexpressed GFP-Dri1-∆C displays either diffuse signal or 1-2 GFP foci. Unlike the full-length Dri1, these GFP foci are immobile.

## Description

Liquid-liquid phase separation (LLPS) of proteins has been implicated in a variety of biological processes, such as formation of membrane less organelles and transcriptional regulation (Hnisz *et al.* 2017; Shin and Brangwynne 2017). LLPS can drive compartmentalization that concentrates biochemical reactions within cells. Heterochromatin is a transcriptionally silent chromatin domain in eukaryotes that is essential for genome stability and gene expression regulation. HP1, a conserved heterochromatin protein, in *Drosophila* and human cells has been shown to exhibit liquid-like phase-separated behavior (Larson *et al.* 2017; Strom *et al.* 2017). But the role of LLPS in heterochromatin formation remains to be elucidated. The fission yeast *Schizosaccharomyces pombe* has emerged as an excellent model system for the study of heterochromatin formation (He *et al.* 2014). We recently showed that a RNA-binding protein Dri1 plays an important role in heterochromatin assembly in fission yeast (Ban *et al.* 2021). Dri1 physically associates with heterochromatic transcripts, and is required for the recruitment of the RNA-induced transcriptional silencing (RITS) complex. Furthermore, Dri1 contributes to the association of the Sir2 histone deacetylase with heterochromatin (Ban *et al.* 2021). In addition to the RNA recognition motif (RRM), Dri1 contains an intrinsically disordered region (IDR) at its C-terminus, which is shared by many proteins undergoing LLPS (Shin and Brangwynne 2017). The C-terminus of Dri1 also contains three zinc fingers. Deletion of the disorder domain or zinc fingers results in defects in heterochromatin silencing, indicating that they are important for the role of Dri1 in heterochromatin silencing (Ban *et al.* 2021). Both IDR and zinc fingers have been shown to be able to drive protein phase separation (Schwartz *et al.* 2013; Shin and Brangwynne 2017; Maharana *et al.* 2018). However, whether Dri1 has the liquid-liquid phase separation property has not been demonstrated. To determine whether Dri1 can undergo LLPS *in vitro* , we fused the different fragments of Dri1 with GFP and test their liquid-droplet formation. Full-length Dri1 fused with GFP (Dri1-GFP) formed solid-like precipitation under physiological conditions (150 mM NaCl, pH7.0-8.0). On the contrary, the C-terminus of Dri1 including three zinc fingers and the IDR with GFP (Dri1-C-GFP) displayed liquid-like phase separation under the same condition. Brightly fluorescent, micrometer-sized droplets are visible for Dri1-C-GFP by fluorescence microscopy (Fig. 1A). In addition, our time-lapse fluorescence microscopy analysis revealed that the spherical droplets in the solution can flow and fuse (Fig. 1B). Thus, Dri1-C-GFP can undergo LLPS *in vitro* . To further investigate how intracellular crowding may affect LLPS property of Dri1, we examined Dri1-C-GFP in the presence of crowding agents, inert macromolecules used to study crowding, including 10% Dextran or Ficoll. We found that Dri1-C-GFP also underwent LLPS in crowders tested, and the number of droplets is substantially increased (Fig. 1C), suggesting that the crowding agents promote LLPS of Dri1 *in vitro* . We also tested whether Dri1 has liquid-like phase-separated behavior *in vivo.* We have previously shown that the signal of GFP-Dri1 expressed at its endogenous level in fission yeast is very weak (Ban *et al.* 2021). We therefore constructed GFP-Dri1 under the thiamine-repressible *nmt1* promoter, and lowly overexpressed GFP-Dri1 by supplementing with 0.05 mM thiamine for 18 hours. We previously showed that lowly overexpressed GFP-Dri1 formed distinct foci mostly in the nucleus (Ban *et al.* 2021). Careful examination of the GFP-Dri1 signal revealed that these foci are usually spherical, a typical shape formed for proteins having liquid-like state (Fig. 1D). Our time-lapse microscopy analysis further revealed that the GFP-Dri1 foci were very dynamic in movement, and can frequently fuse together (Fig. 1D), suggesting that Dri1 has liquid-liquid phase separation properties *in vivo.* Furthermore, we deleted the C-terminus of Dri1, which contains the IDR region and three zinc fingers (Dri1 *-∆C* ). We found that lowly overexpression of GFP-Dri1-∆C results in few immobile foci or dispersed GFP signal, indicating that the liquid phase separation property is lost in the mutant (Fig. 1D). It has been shown that overexpression of proteins with LLPS property impairs cell growth (Bolognesi *et al.* 2016). In agreement with this, we also showed that that strong overexpression of Dri1 results in severe growth defects (Ban *et al.* 2021). Our data thus demonstrate that C-terminus of Dri1 displays liquid-like property, which contributes to its role in heterochromatin silencing. We propose that the phase separated condensates formed by Dri1 may help recruit RNAi components and HDAC to mediate heterochromatin assembly.

## Methods

**Media and DNA constructs**

Standard media for fission yeast growth were used (Moreno *et al.* 1991). Full-length Dri1 and C-terminus-deleted Dri1 (Dri1-∆C) were cloned into the pREP1 vector containing in-frame GFP under the *nmt1* promoter. The constructs were lowly overexpressed by supplementing the medium with 0.05 mM thiamine for 18 hours.

**Protein expression and purification**

Dri1 fragments were cloned into a modified pET28b vector with a 6xHis-SUMO protein fused at the N terminus and a GFP protein fused at the C terminus, and were expressed in Rosetta cells. Dri1 fragments were purified by using Ni-NTA agarose beads (Qiagen). Ulp1 protease was subsequently added to remove the N-terminal 6xHis-SUMO tag, and Dri1-GFP proteins were collected after ULP1 on-beads digestion. The proteins were further purified by gel-filtration chromatography on Hiload Superdex 200 column (GE Healthcare).

***In vitro* phase separation assays**

For the droplet formation, proteins were diluted to the final concentration (30 mM) with the phase separation buffer (25mM Tris-HCl pH 8.0,150 mM NaCl, 0.2mM DTT) in 0.2 ml PCR tubes. In experiments using Dextran-70 or Ficoll 400 as crowding agents, crowders were added to reach the final concentrations (10%) in the phase separation buffer. Fluorescent images were acquired on a Zeiss LSM 710 microscope with a 63x objective.

**Microscopic analysis**

Microscopic analysis was performed as described (Dong *et al.* 2016). Briefly, fluorescence signal was captured by DeltaVision system (Applied Precision, Issaquah, WA). Images were taken as z-stacks of 0.2-µm increments with an oil immersion objective (×100).

## Reagents

All the *in vivo* experiments were performed using the haploid strains of *S. pombe* (FL 255 *h ^-^ leu1-32 ura4-D18 ade6-216 his3-D1* ).
